# Caso de envenenamiento por picadura de pez león, atendido en un servicio de urgencias de Colombia

**DOI:** 10.7705/biomedica.7230

**Published:** 2025-08-11

**Authors:** María Alejandra Montoya-Giraldo, Sebastián Franco González, Andrés F. Zuluaga

**Affiliations:** 1 Laboratorio Integrado de Medicina Especializada, Hospital Alma Mater de Antioquia, Facultad de Medicina, Universidad de Antioquia, Medellín, Colombia Universidad de Antioquia Universidad de Antioquia Medellín Colombia; 2 Oficina de Coordinación, Unidad Funcional de Urgencias, Los Cobos Medical Center, Bogotá, D. C., Colombia Los Cobos Medical Center Los Cobos Medical Center Bogotá, D. C. Colombia

**Keywords:** intoxicación, animales ponzoñosos, peces venenosos, ambiente marino, accidente, Colombia, Envenomation, animals, poisonous, fishes, poisonous, marine environment, accidents, Colombia

## Abstract

El pez león (*Pterois volitans*) es una especie invasora del mar Caribe que puede inyectar veneno por medio de sus espinas. Se informa un caso de este tipo de accidente, que podría convertirse en un riesgo emergente para la salud pública en Colombia. Se trata de una mujer sana de 59 años que sufrió una lesión por pinchazo con la aleta de un pez león en el cuarto dedo de la mano izquierda mientras buceaba en Aruba.

La paciente fue tratada en el lugar del accidente con compresas calientes, antihistamínicos y corticoides sistémicos; no obstante, experimentó dolor intenso, frialdad y palidez en todo el dedo afectado.

Al día siguiente, la paciente viajó a Bogotá (Colombia) y, posteriormente, presentó edema, pérdida de la sensibilidad y cambios en la coloración de la piel. En el servicio de urgencias donde consultó, se administró un tratamiento farmacológico con antiagregante plaquetario (100 mg de ácido acetilsalicílico por vía oral), anticoagulante (60 mg de enoxaparina por vía subcutánea) y un antagonista del calcio para controlar el vasoespasmo (30 mg de nifedipino por vía oral); además, se solicitaron estudios paraclínicos complementarios. Estos exámenes y la ecografía Doppler de los vasos arteriales del miembro superior izquierdo, fueron normales. El tratamiento fue exitoso y la paciente se recuperó por completo.

Dada la novedad del caso en Colombia y su evolución favorable, a pesar de las limitaciones del tratamiento inicial y de lo tardío del farmacológico, se presenta una discusión amplia basada en la revisión de la literatura disponible.

## Abstract (en)

The lionfish (*Pterois volitans*) is an invasive species in the Caribbean Sea that can accidentally inject venom into humans through its spines. Here, we report a case of lionfish envenomation, highlighting a potential emerging threat to public health in Colombia. The case describes a healthy 59-year-old woman who suffered an accidental sting on the fourth finger of her left hand after contact with a lionfish spin while diving in Aruba.

On-site treatment included hot compresses, antihistamines, and systemic corticosteroids. However, the patient experienced severe pain, coldness, and paleness in the affected finger. One day later, the patient traveled to Bogotá, Colombia, and developed swelling, loss of sensitivity, and changes in skin coloration. In the emergency unit, the patient received pharmacological treatment with 100 mg of aspirin administered orally and 60 mg of enoxaparin administered subcutaneously; also, a calcium antagonist was prescribed for vasospasm management (30 mg of nifedipine 30 administered orally), and complementary studies were ordered. Paraclinical results were normal, and the Doppler ultrasound of the left upper limb arterial vessels showed no abnormalities. The treatment was successful, and the patient fully recovered.

Given the novelty of this affection for our country, the patient’s favorable evolution after latestage pharmacological management, and the limitations of the initial treatment, we present a broad discussion based on the review of related literature.

*Pterois volitans*, comúnmente llamado pez león colorado, es uno de los peces venenosos de la familia Scorpaenidae. Originario de los arrecifes de coral de los océanos Índico y Pacífico occidental, este pez ha invadido el océano Atlántico y el mar Caribe y, además, se vienen utilizando como peces ornamentales en acuarios de Latinoamérica. Aunque más del 50 % de todos los vertebrados venenosos son peces, la expansión global de esta especie depredadora y venenosa la ha posicionado como una amenaza importante para los buceadores, quienes describieron su aparición en la década de los noventa, en las costas de Florida, Estados Unidos. Desde entonces, se ha reportado su diseminación en las Bahamas (2004), Cuba (2007), y el archipiélago de San Andrés y Providencia (2008) ^1^.

Aunque se han reportado accidentes con peces león, principalmente en Cuba ^2^ y Honduras ^3^, no se ha descrito ninguno atendido en Colombia y, menos, con tratamiento tardío. Algunos autores han acuñado el término “lesiones emergentes” para describir la aparición de este tipo de accidentes debido a la introducción de una especie invasora en nuevas regiones geográficas ^1^.

Se presenta el caso de una mujer sana de 59 años que sufrió una lesión por pinchazo con la aleta de un pez león en el cuarto dedo de la mano izquierda, mientras buceaba en Aruba; posteriormente, presentó dolor, cambio de color y pérdida de sensibilidad en el dedo de la mano afectada, mientras regresaba a Bogotá. Se detallan los hallazgos clínicos durante la atención del incidente, las limitaciones presentadas, los resultados de laboratorio, el manejo y la evolución exitosa de la paciente. Además, se revisa y discute la literatura científica disponible sobre el tema.

## Descripción del caso

Una mujer de 59 años, psicóloga, sin antecedentes patológicos conocidos, sufrió una picadura accidental con la aleta de un pez león en el cuarto dedo de la mano izquierda mientras buceaba en Aruba. El animal fue identificado fácilmente debido a sus conocidas características morfológicas.

Durante el accidente, la paciente experimentó un intenso nerviosismo y describió sensaciones de desesperación debido a la intensidad del dolor y a la progresiva pérdida de sensibilidad en el dedo afectado. Al regresar a tierra firme, además del dolor intenso, la paciente presentó frialdad y palidez en la zona comprometida. Estos síntomas se trataron *in situ* con compresas calientes y con la administración de un antihistamínico y corticoides sistémicos, lo que proporcionó una aparente mejoría.

Un día después, la mujer viajó a Bogotá -48 horas después del accidente-, persistían los cambios en la coloración de la piel, además de la aparición de edema y la pérdida de sensibilidad en la zona afectada. Por esta razón, la paciente decidió acudir a la Unidad Funcional de Urgencias de Los Cobos Medical Center en Bogotá.

**Figura 1:**
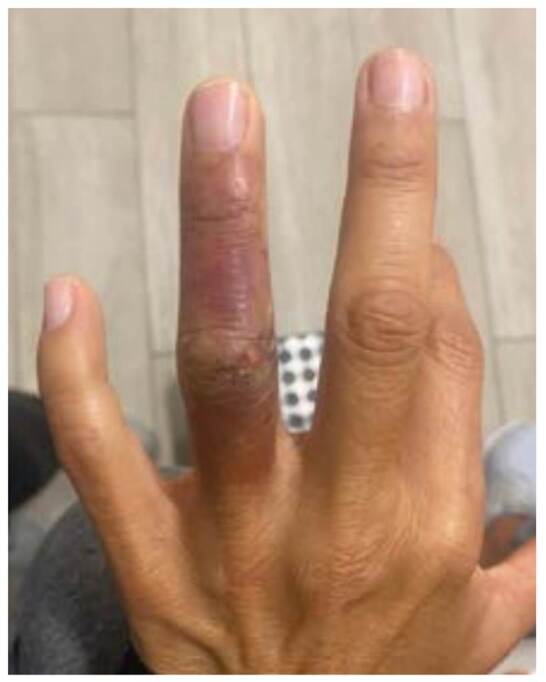
Aspecto de la zona afectada y herida en el dorso de la articulación interfalángica proximal del dedo anular de la mano izquierda al ingreso de la paciente al servicio de urgencias de Los Cobos Medical Center en Bogotá, Colombia.

En el momento de su ingreso a urgencias, la paciente presentaba signos vitales dentro de los rangos normales: presión arterial de 110/70 mm Hg, frecuencia cardíaca de 72 latidos por minuto, frecuencia respiratoria de 14 respiraciones por minuto y temperatura corporal de 36,8 °C.; manifestaba dolor y presentaba coloración violácea del cuarto dedo de la mano izquierda, con frialdad local y perfusión distal lenta, sin sensibilidad ni evidencia de ningún cuerpo extraño o secreción purulenta (figura 1).

Dados los hallazgos clínicos de compromiso de la perfusión y la sensibilidad del dedo afectado, se administró un tratamiento farmacológico con antiagregante plaquetario (100 mg de ácido acetilsalicílico por vía oral), anticoagulante (60 mg de enoxaparina por vía subcutánea) y un antagonista del calcio para controlar el vasoespasmo (30 mg de nifedipino por vía oral); además, se solicitaron estudios paraclínicos complementarios.

Los resultados de los exámenes de laboratorio solo revelaron leucopenia leve y se resumen en el cuadro 1. En la ecografía Doppler de vasos arteriales del miembro superior izquierdo se apreciaron vasos de calibre y trayecto normales, con paredes delgadas, regulares, sin cambios ateroscleróticos significativos y con un patrón de flujo normal, sin estenosis ni obstrucción. Tampoco, se observaron aneurismas, fístulas arteriovenosas o trombosis.

**Cuadro 1:** Resultados de los exámenes de laboratorio obtenidos al ingreso del paciente al servicio de urgencias

Parámetro	Resultado	Valores de referencia
Leucocitos (células/μΙ)	4.100	4.500-11.000
Hemoglobina (g/dl)	15	12-16
Hematocrito (%)	43,2	36-47
Plaquetas (células/μΙ)	227.000	150.000-450.000
Creatinina (mg/dl)	0,87	0,7-1,3
Nitrógeno ureico en sangre (mg/dl)	19,3	6-24
Proteína C reactiva (mg/L)	1,79	< 3
Velocidad de sedimentación globular (mm/h)	7	< 20
Tiempo de protrombina (s)	11,2	10-13
Índice internacional normalizado (INR)	0,89	0,8-1,2
Tiempo parcial de tromboplastina (s)	22,8	22-35

Dada la ausencia de un síndrome de reacción inflamatoria sistémica -sin elevación de reactantes de fase aguda, alteraciones de la función renal o los tiempos de coagulación, y con un Doppler arterial dentro de los parámetros normales- se le ofreció la posibilidad de practicar un bloqueo nervioso en la base del dedo como opción para controlar el dolor. El bloqueo digital se ha recomendado en este tipo de accidentes para proporcionar un alivio efectivo y duradero en casos de dolor grave que no responden adecuadamente al tratamiento convencional ^4^^,^^5^. Esta técnica busca interrumpir la transmisión de señales de dolor a los nervios periféricos afectados y prevenir complicaciones asociadas, como el vasoespasmo prolongado.

**Figura 2:**
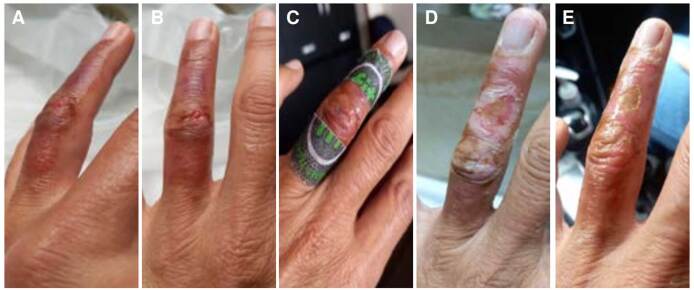
Evolución de la lesión en la mano izquierda tras el envenenamiento por la mordedura de un pez león: A) vista oblicua a las 72 horas después del accidente; B) vista dorsal a las 72 horas después del accidente; C) vista dorsal a las 120 horas después del accidente; estado de la lesión a los 7 (D) y 15 (E) días después del manejo de la herida en el servicio de urgencias.

Sin embargo, la paciente rechazó esta opción terapéutica y fue dada de alta con manejo de la lesión en casa mediante antibioticoterapia (500 mg de cefalexina por vía oral, cada 6 horas durante 7 días) y el antagonista del calcio (30 mg de nifedipino por vía oral, cada 8 horas durante 5 días). Se inició manejo complementario con parches de terapia con tecnología de infrarrojo lejano (*Far Infrared Technology*, FIT) para controlar el dolor ^6^. Se recomendó control ambulatorio por la especialidad de cirugía de mano y seguimiento telefónico. A las 24 horas, la paciente solo refirió sensación de ardor (figura 2A y 2B).

A las 96 horas, la paciente fue valorada por un especialista en cirugía de mano, quien evidenció edema y una zona de quemadura química sobre el dorso del dedo, con buen llenado capilar e hipoestesia. No se realizaron cambios en el tratamiento. A las 120 horas, durante el seguimiento telefónico, la paciente refirió dolor leve de tipo ardor, recuperación progresiva de la sensibilidad en la falange distal e inicio de cicatrización de la herida (figura 2C).

Al séptimo día de seguimiento, la paciente reportó mejoría clínica del dolor y continuación del proceso de cicatrización de la herida del cuarto dedo de la mano izquierda, con mejoría del llenado capilar, leve disminución de la sensibilidad (hipoestesia) distal y sin limitación de la movilidad (figura 2D).

Dada la evolución clínica favorable, se suspendió el antibiótico y se indicó curación cada tres días con apósito de hidrofibra de hidrocoloide y nitrofurazona. Se dieron recomendaciones, se señalaron los signos de alarma y se dio de alta. A los 15 días de seguimiento del accidente, la paciente presentaba una mejoría casi completa de la lesión en la mano (figura 2E).

### Consideraciones éticas

Se obtuvo el consentimiento informado de la paciente, el cual incluyó la garantía de la confidencialidad en la información personal y la autorización para usar las fotografías tomadas a la lesión.

## Discusión

El pez león se ha convertido en una de las especies invasoras más amenazantes para la vida en los arrecifes de coral, con una distribución que se extiende desde los estados del Atlántico de Estados Unidos hasta Brasil, incluyendo todo el mar Caribe ^1^.

Por su amplia distribución, este pez puede afectar la salud humana, al menos, de dos maneras: (i) por el consumo de especímenes que han acumulado ciguatoxinas liposolubles, lo que produce una intoxicación alimentaria conocida como ciguatera ^7^, y (ii) por la posibilidad de que su picadura resulte en un envenenamiento grave, especialmente, entre acuariófilos ^4^^,^^8^, practicantes de deportes náuticos cerca de los arrecifes de coral y buzos que están en riesgo de contacto directo de dedos y manos por manipulación del pez en jornadas de caza comercial o de salud ambiental para erradicar este invasor, o de forma indirecta, cuando entran en contacto con las espinas remanentes del pez en tanques o aletas.

Aunque existen dos especies invasoras, *P*. *volitans* y *P*. *miles*, la primera es la predominante en el mar Caribe y se caracteriza por poseer espinas en sus aletas dorsal, pélvica y anal. Estas espinas están recubiertas por glándulas que liberan veneno cuando se rompen mecánicamente tras el contacto con la víctima. La composición del veneno de esta especie en territorio colombiano fue descrita recientemente por Pérez-Bravo ^9^. En dicho análisis preliminar, el autor encontró una gran cantidad de proteínas de alto y bajo peso molecular, como las halladas en otros peces venenosos. Entre las más relevantes, se encuentran aquellas con actividad hemolítica (hemolisinas con peso molecular de 43 kDa) y aglutinante de eritrocitos mediada por lectinas entre 10 y 45 kDa; asimismo, una lectina que reconoce manosa, proteasas de sustratos como gelatina y caseína, actividad de hialuronidasa y fosfolipasas ^9^.

Las manifestaciones locales del envenenamiento por pez león suelen incluir una herida punzante acompañada de eritema, edema, palidez o equimosis, y dolor intenso que, en ocasiones, puede irradiarse a las regiones proximales. También, se han documentado síntomas como anestesia, parestesia y linfedema asociados con la lesión ^8^^,^^10^. La acción de la hialuronidasa y las fosfolipasas presentes en el veneno del pez león, puede alterar la matriz extracelular y los fosfolípidos de las membranas celulares, lo que contribuye significativamente a la reacción inmunológica de inflamación y dolor intenso. Aunque los síntomas sistémicos no son comunes en este tipo de envenenamientos, pueden incluir hipotensión o hipertensión, taquicardia o bradicardia y, en algunos casos, fiebre, sudoración fría, síncope, náuseas, vómitos, disnea y convulsiones.

Aunque no se han reportado muertes directamente relacionadas con el envenenamiento por pez león, existe la posibilidad teórica de una reacción anafiláctica fatal en casos graves ^11^^,^^12^.

El número de accidentes ha venido en ascenso y los reportes de casos por fuera de Colombia no son escasos. Por ejemplo, Resiere *et al*., hicieron un estudio prospectivo con 117 afectados en dos años ^12^, quienes manifestaron principalmente dolor, edema, parestesias y dolor abdominal. Los primeros tres síntomas también se presentaron en este caso. Haddad Jr. *et al*., ^11^ describieron en Brasil 15 casos de envenenamiento por pez león entre 1997 y el 2014, y reportaron que los cambios de coloración en la piel -incluso la cianosis-, fue evidente en todos ellos, lo que refuerza la importancia de manejar adecuadamente la vasoconstricción de la zona afectada como parte del control de la sintomatología ^11^. En este caso, este hecho propició el uso de nifedipino.

De hecho, la administración de nifedipino, aspirina y heparina de bajo peso molecular fue novedosa y anteriormente no se había reportado como parte del tratamiento de los pacientes que sufren este tipo de accidentes. En este estudio, se consideró que el uso de los tres medicamentos estaba justificado por el mecanismo de acción de las toxinas del pez león y el desenlace favorable de la paciente. Algunos autores describen que las manifestaciones clínicas pueden tardar hasta 30 días en mejorar por completo ^12^.

En el estudio de Mouchbahani-Constance *et al*., se analizaron -*in vitro* e *in vivo*- los mecanismos mediante los cuales las toxinas del pez león ocasionaban dolor intenso ^13^. Estos autores encontraron que los compuestos algogénicos del pez son péptidos termolábiles que causan inflamación neurogénica en el sitio de la inyección y que inducen la vía de señalización de FOS para la subsecuente activación de la microglía en las capas superficiales del asta dorsal. De hecho, Latremoliere y Woolf reportaron que el veneno actúa predominantemente en nociceptores no peptidérgicos y el TRPV1 negativo (receptor vaniloide). Estos nocirreceptores expresan canales de calcio dependientes del voltaje que, al activarse, generan efectos hiperalgésicos y, por tanto, pueden ser bloqueados selectivamente con diltiazem y nifedipino ^14^.

El veneno tiene un reconocido potencial citotóxico, proinflamatorio y protrombótico, propiedades que pueden ameritar una intervención con un agente antitrombótico ^12^. También, se ha descrito el uso de enoxaparina cuando el daño de la matriz extracelular por toxinas pueda propiciar respuestas inmunológicas que desencadenen microangiopatías ^15^.

En varios reportes de la literatura médica, se recomienda, como medida general, sumergir la zona afectada en agua caliente para controlar el dolor durante las primeras horas del accidente, cosa que no sucedió en este caso. En este sentido, la temperatura del agua debe oscilar entre los 40 y los 45 °C, sin exceder ese rango. Diferentes autores reconocen que el agua caliente destruye las proteínas termolábiles del veneno ^9^^,^^13^^,^^16^ y favorecen el control óptimo de la intoxicación ^16^. En el estudio de Resiere *et al*. ^12^, se observó que la persistencia de dolor por más de 24 horas estaba asociada a erupción cutánea y parestesias, así como con otras complicaciones. A quienes se les aplicó agua caliente en las tres primeras horas, tuvieron un control del dolor significativamente mayor en las primeras 24 horas.

Ya que esta es una medida de primeros auxilios y, además, económica, debería reforzarse su uso en todos los casos. También, se puede aplicar anestesia local o regional si el dolor no mejora con el agua caliente ^4^^,^^10^. Se prefiere la bupivacaína debido a su acción prolongada ^17^. Sin embargo, en este caso, la paciente rechazó este tratamiento.

Se requiere profilaxis antitetánica en todos los casos y, aunque no hay consenso al respecto, algunos autores recomiendan administrar antibióticos en envenenamientos por pez león o pez piedra ^10^.

## Conclusión

La intoxicación por pez león se está convirtiendo en una lesión emergente cada vez más frecuente en los países que limitan con el océano Atlántico. Aunque el accidente reportado ocurrió en Aruba, este tipo de envenenamiento puede representar un riesgo creciente en las costas colombianas debido a la presencia del pez león en la región.

Para evitar una evolución tórpida de la intoxicación es crucial que los bañistas e individuos expuestos a peces venenosos reciban el tratamiento adecuado desde el principio, lo que incluye la aplicación local de calor. En el ámbito médico, aunque la mayoría de los casos tienden a ser favorables, es importante tener en cuenta que la atención tardía puede requerir el uso de fármacos anticoagulantes, antiagregantes y vasodilatadores para controlar los síntomas asociados.

Es fundamental aumentar la conciencia pública sobre esta lesión emergente y mejorar el manejo clínico, para minimizar su impacto en la salud pública.
